# High Magnetic Field Sensitivity in Ferromagnetic–Ferroelectric Composite with High Mechanical Quality Factor

**DOI:** 10.3390/s20226635

**Published:** 2020-11-19

**Authors:** Yong-Woo Lee, Joon-Young Soh, Il-Ryeol Yoo, Jiung Cho, Cheol-Woo Ahn, Jong-Jin Choi, Byung-Dong Hahn, Kyung-Hoon Cho

**Affiliations:** 1 School of Materials Science and Engineering, Kumoh National Institute of Technology, Gumi 39177, Korea; dyddnsub@kumoh.ac.kr (Y.-W.L.); yiy1204@kumoh.ac.kr (I.-R.Y.); 2 New & Renewable Energy Lab., KEPCO Research Institute, Daejeon 34056, Korea; kepsoh@kepco.co.kr; 3 Western Seoul Center, Korea Basic Science Institute, Seoul 03579, Korea; 4 Korea Institute of Materials Science (KIMS), Changwon 51508, Korea; cheoruahn@kims.re.kr (C.-W.A.); finaljin@kims.re.kr (J.-J.C.); cera72@kims.re.kr (B.-D.H.)

**Keywords:** magnetoelectric, ferroelectric, piezoelectric, magnetic field, composites

## Abstract

In this study, composite devices were fabricated using ferromagnetic FeSiB-based alloys (Metglas) and ferroelectric ceramics, and their magnetic field sensitivity was evaluated. Sintered 0.95Pb(Zr_0.52_Ti_0.48_)O_3_-0.05Pb(Mn_1/3_Sb_2/3_)O_3_ (PZT-PMS) ceramic exhibited a very dense microstructure with a large piezoelectric voltage coefficient (*g*_31_ = −16.8 × 10^−3^ VmN^−1^) and mechanical quality factor (*Q*_m_ > 1600). Owing to these excellent electromechanical properties of the PZT-PMS, the laminate composite with a Metglas/PZT-PMS/Metglas sandwich structure exhibited large magnetoelectric voltage coefficients (*α*_ME_) in both off-resonance and resonance modes. When the length-to-width aspect ratio (*l*/*w*) of the composite was controlled, *α*_ME_ slightly varied in the off-resonance mode, resulting in similar sensitivity values ranging from 129.9 to 146.81 VT^−1^. Whereas in the resonance mode, the composite with small *l*/*w* exhibited a large reduction of *α*_ME_ and sensitivity values. When controlling the thickness of the PZT-PMS (*t*), the *α*_ME_ of the composite showed the largest value when *t* was the smallest in the off-resonance mode, while *α*_ME_ was the largest when *t* is the largest in the resonance mode. The control of *t* slightly affected the sensitivity in the off-resonance mode, however, higher sensitivity was obtained as *t* increased in the resonance mode. The results demonstrate that the sensitivity, varying with the dimensional control of the composite, is related to the mechanical loss of the sensor. The composite sensor with the PZT-PMS layer exhibited excellent magnetic field sensitivity of 1.49 × 10^5^ VT^−1^ with a sub-nT sensing limit, indicating its potential for application in high-performance magnetoelectric sensor devices.

## 1. Introduction

Magnetoelectric (ME) composites comprising ferroelectric (FE) and ferromagnetic (FM) materials have the advantageous property of being capable of converting magnetic fields into electric fields and vice versa [1,2,3]. This field conversion, known as the ME effect, is possible through a strain coupling between the FE and FM constituents of the composites. For the direct ME effect, the FM material generates a mechanical strain when an external magnetic field varies (by magnetostriction, converse piezomagnetic effect), and the strain is transferred to the poled FE material at the FM/FE interface, thus generating a potential difference (by a direct piezoelectric effect). Strain-mediated coupling in a composite depends on the interfacial coupling between the two ferroic phases of the composite and can be optimized via microstructural designs [4] as well as controlling the physical properties of interfacial bonding materials [5,6]. Based on the ME effect, various electronic device applications, such as magnetic field sensors, current sensors, gyrators, position sensors, energy harvesters, resonators, and filters, have been proposed [1,7,8,9,10,11].

Research on ME composites for magnetic field sensors is of technological importance because ME composites have a simple structure and require a facile fabrication process despite their good field conversion performance. The magnetic field to electric field conversion performance is usually evaluated via the *ME voltage coefficient* (*α*_ME_), which can be given by the following expression:

(1) $$\alpha_{\mathrm{ME}}=\frac{{dE}_{\mathrm{AC}}}{{dH}_{\mathrm{AC}}}=\frac{{dV}_{\mathrm{AC}}}{{dH}_{\mathrm{AC}}}\times \frac{1}{t_{p}},$$

where *E*_AC_ is the output electric field, *H*_AC_ is the input magnetic field strength, *V*_AC_ is the output potential difference, and *t*_p_ is the distance between the two electrodes of the FE phase. The term d*V*_AC_/d*H*_AC_ in Equation (1) determines the magnetic field sensitivity and reflects the precision with which a magnetic field change can be detected using a voltmeter. Therefore, it is important to enhance the magnitude of *α*_ME_ as much as possible to obtain a high magnetic field sensitivity.

As the ME effect in ME composites is a product property of the FM and FE phases, *α*_ME_ is represented by the following equation, which is based on the inherent material properties of the individual FM and FE phases [11,12,13]:

(2) $$\alpha_{\mathrm{ME}}=\frac{nqg}{{nS}^{E}\left(1-k^{2}\right)+\left(1-n\right)S^{H}}\times {\left[\exp\left(\tan\;\delta +\tan\;\theta +\frac{C-C_{f}}{C_{f}}\right)\right]}^{-1},$$

where *n* is the volume fraction of the FM phase; *q* is the piezomagnetic coefficient of the FM phase; *g* and *k* are the piezoelectric voltage coefficient and electromechanical coupling coefficient of the FE phase, respectively; *S*^E^ and *S*^H^ are the elastic compliances of the FE and FM phases, respectively; tan *δ* is the dielectric loss; tan *θ* is the piezoelectric loss; *C* is the capacitance at a given frequency; *C_f_* is the free capacitance. Therefore, the selection of an FE material with a large *g* value and small tan *δ* value and an FM material having a large *q* value is favorable for realizing a large *α*_ME_. Moreover, a high mechanical quality factor (*Q*_m_) of the FE phase is important for enhancing the *α*_ME_ in the resonance mode of ME devices [14,15,16].

ME magnetic field sensors that comprise FM single crystals have been investigated owing to their excellent piezoelectric properties, demonstrating nT- to fT-level sensing limits [17,18,19]. These encouraging results demonstrate the feasibility of implementing small and efficient ME magnetic field sensor devices, which are comparable to conventional search coil and superconducting quantum interference device sensors [20] but do not require complicated fabrication processes or special equipment. However, the application of polycrystalline FE ceramics fabricated via an economical ceramic process would be more desirable from an industrial viewpoint, i.e., for mass production and to realize inexpensive sensor devices.

In this study, we employed polycrystalline FE materials to evaluate the magnetic field sensing performance of an ME composite comprising polycrystalline FE materials. A 0.25 wt% PbO-added 0.95Pb(Zr_0.52_Ti_0.48_)O_3_-0.05Pb(Mn_1/3_Sb_2/3_)O_3_ (PZT-PMS) ceramic was synthesized, and a commercial PZT5A ceramic was prepared for comparison. An FeSiB-based amorphous alloy (Metglas) was selected as the FM material. First, the electromechanical characteristics of the FE materials and the *α*_ME_ values of the FM/FE/FM laminate composites were measured and analyzed. The magnetic field sensitivity and sensing limit of the ME composites were then evaluated. We demonstrate that the sensitivity is related to the piezoelectric voltage coefficient of the FE layer in the off-resonance mode, while the sensitivity of the resonance mode is predominantly affected by the mechanical quality factor of the composite sensor. Furthermore, we discuss the effect of dimensional control of the FE layer on the magnetic field sensing performance of the PZT-PMS sensors.

## 2. Materials and Methods

Reagent-grade raw powders of PbO, ZrO_2_, TiO_2_, MnO_2_, and Sb_2_O_3_ (all from Kojundo Chemical Lab. Co., Sakado, Japan) were mixed in accordance with the stoichiometric composition of the PZT-PMS and ball-milled with ethyl alcohol and yttria-stabilized zirconia balls in a polyethylene jar for 24 h. The powder mixture was dried at 80 °C for 24 h and subsequently calcined at 850 °C for 2 h in air. The calcined PZT-PMS powder was ball-milled again for 24 h with 0.25 wt% PbO powder and dried at 80 °C for 24 h. The dried powder was unidirectionally pressed into a rectangular-shaped compact under 100 MPa, and the powder compact was sintered at 1240 °C for 8 h in air. The sintered PZT-PMS samples were cut into 31-mode rectangular plates of (10.5, 12.0, 13.5 mm) (*l*) × 3.0 mm (*w*) × (0.5, 1.0, 1.5 mm) (*t*). A silver electrode was deposited at the top and bottom surfaces of the PZT-PMS plates and the poling process was performed by applying an electric field of 3 kVmm^−1^ for 5 min at 120 °C in silicone oil. For performance comparison, a commercial PZT5A (PSI-5A4E, Piezo Systems, Inc., Cambridge, MA, USA) ceramic plate of 13.5 mm (*l*) × 3.0 mm (*w*) × 1.0 mm (*t*) was prepared. The microstructure and phase formation of the sintered PZT-PMS sample were examined by scanning electron microscopy (SEM) (JSM-6500F, JEOL, Ltd., Akishima, Tokyo, Japan), energy-dispersive X-ray spectroscopy (EDS) (XFlash 630, Bruker Nano GmbH, Berlin, Germany), and X-ray diffraction (XRD) (D-MAX/2500, Rigaku Co., Tokyo, Japan). The bulk density of the polished 31-mode samples was measured employing the Archimedes method. The impedance and phase angle spectra and piezoelectric and dielectric properties of the pooled samples were measured using an impedance analyzer (IM3570, Hioki EE Co., Nagano, Japan).

The FM/FE/FM laminate composites were fabricated by attaching 90 μm-thick Metglas sheets (2605SA1, Metglas Inc., Conway, SC, USA) at the top and bottom surfaces of the poled PZT-PMS plates and PZT5A plate using an epoxy adhesive (DP460, 3M Company, St. Paul, MN, USA). The ME voltage coefficient as a function of the DC magnetic field strength (*H*_DC_) and *H*_AC_ frequency of the laminate composites were measured using a Helmholtz coil, electromagnet, and lock-in amplifier (SR860, Stanford Research Systems, Sunny-vale, USA) [13]. To evaluate the magnetic field sensitivity and sensing limit of the laminate composites, the output voltage was monitored as a function of the *H*_AC_. The *H*_AC_ was controlled by the Helmholtz coil, and the output voltage from the laminate composite was measured using a lock-in amplifier.

## 3. Results and Discussion

**Figure 1:**
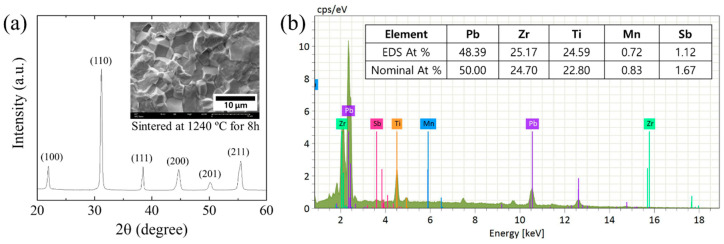
(**a**) XRD pattern of PZT-PMS ceramic sample sintered at 1240 °C for 8 h. Inset image presents an SEM microstructure image of the sintered PZT-PMS sample. (**b**) Energy-dispersive X-ray spectroscopy (EDS) result of the sintered PZT-PMS ceramic sample.

Figure 1a confirms that a perovskite phase was well-formed (JCPDS 01-070-4265) without secondary phases in a 0.25 wt% PbO-added PZT-PMS ceramic sample sintered at 1240 °C for 8 h in air. The sintered PZT-PMS sample exhibited a very dense microstructure comprising faceted grains with an average grain size of 4 μm, as presented in the inset image of Figure 1. The bulk density of the PZT-PMS sample was measured as 7.931 gcm^−3^, which is almost 99% of the theoretical density. The EDS result in Figure 1b shows that the elemental composition of the sintered PZT-PMS is closed to the nominal stoichiometric composition.

**Figure 2:**
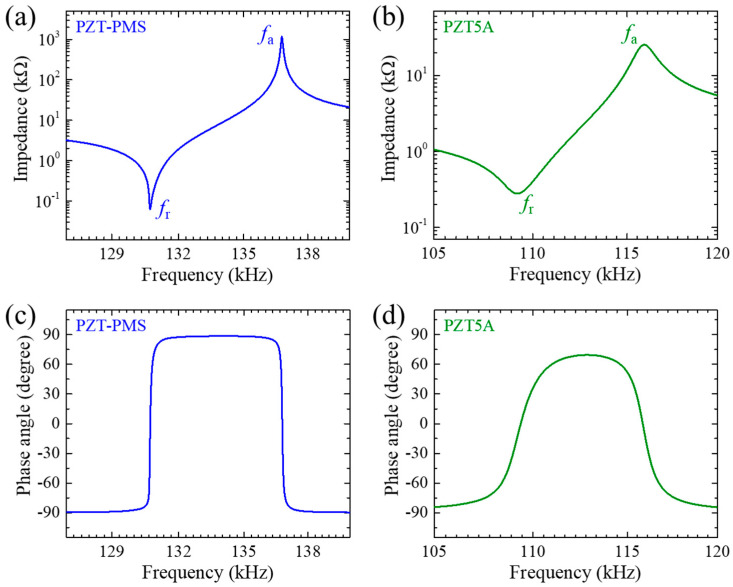
Impedance spectra of 31-mode piezoelectric samples: (**a**) PZT-PMS and (**b**) PZT5A. Phase angle spectra of 31-mode piezoelectric samples: (**c**) PZT-PMS and (**d**) PZT5A.

Figure 2 presents the impedance and phase angle spectra of the poled PZT-PMS and PZT5A samples with 31-mode dimensions (13.5 mm (*l*) × 3.0 mm (*w*) × 1.0 mm (*t*)). The elastic moduli of the PZT-PMS and PZT5A samples were calculated as 100 and 65 GPa, respectively, which resulted in a higher resonance frequency range for the PZT-PMS despite the same dimensions of the two samples. The piezoelectric charge coefficient (*d*_31_), electromechanical coupling coefficient (*k*_31_), and dielectric constant (*ε*_33_^T^/*ε*_0_) of the PZT5A were greater than those of the PZT-PMS, as shown in Table 1, however, the piezoelectric voltage coefficient (*g*_31_) was larger in the PZT-PMS owing to much smaller *ε*_33_^T^/*ε*_0_ value of the PZT-PMS. As observed from Figure 2, the peaks for the *resonance frequency (f*_r_) and anti-resonance frequency (*f*_a_) of PZT-PMS are much sharper than those of PZT5A, and the slope of the *phase angle change* at *f*_r_ and *f*_a_ is greater in PZT-PMS. This indicates that the mechanical loss by power consumption is greater in PZT5A than in the case of PZT-PMS. Accordingly, the mechanical quality factors (*Q*_m_, the inverse of mechanical loss) obtained at both *f*_r_ and *f*_a_ demonstrated a large difference between the two samples, as shown in Table 1.

**Figure 3:**
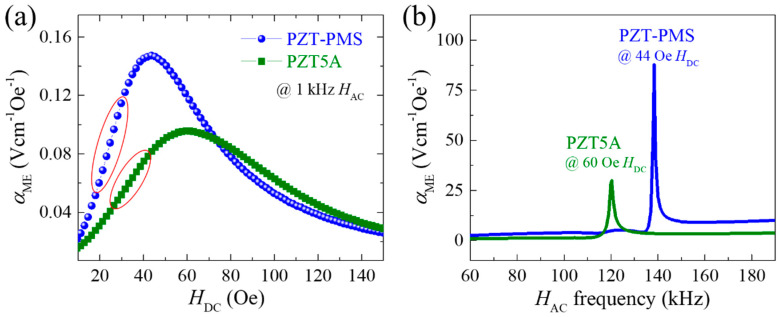
(**a**) Magnetoelectric voltage coefficient of PZT-PMS and PZT5A sensors measured at 1 kHz as a function of *H*_DC_. (**b**) Magnetoelectric voltage coefficient spectra of PZT-PMS and PZT5A sensors as a function of *H*_AC_ frequency. (FE dimensions: 13.5 mm (*l*) × 3.0 mm (*w*) × 1.0 mm (*t*)).

In the direct ME effect, the *α*_ME_ value is enhanced at *f*_a_ of the laminate composite system, and the degree of enhancement is dependent on the *Q*_m_ value of the system [14,15,21]. The *f*_a_ obtained from the composite system is close to that obtained from the FE material when the thickness fraction of the FE layer is sufficiently large [15]. From Equation (2) and the data in Table 1 (*g*_31_, *Q*_m_ at *f*_a_, and tan *δ*), it was clearly expected that the *α*_ME_ value of the laminate composite with PZT-PMS (PZT-PMS sensor) would be superior to that of the laminate composite with PZT5A (PZT5A sensor) both in the resonance and off-resonance modes. As shown in Figure 3a, the composite sensor devices (FE dimensions: 13.5 mm (*l*) × 3.0 mm (*w*) × 1.0 mm (*t*)) exhibited typical *α*_ME_ versus *H*_DC_ curves at a 1 kHz *H*_AC_, with maximum *α*_ME_ values of 0.147 and 0.096 Vcm^−1^Oe^−1^ for the PZT-PMS and PZT5A sensors, respectively. When the *H*_AC_ frequency was tuned to the anti-resonance frequency of the sensors, *α*_ME_ was significantly enhanced, as shown in Figure 3b. For the PZT5A sensor, an *α*_ME_ of 30.1 Vcm^−1^Oe^−1^ (314 times greater than that at 1 kHz) was obtained. The PZT-PMS sensor exhibited a large *α*_ME_ of 87.6 Vcm^−1^Oe^−1^, which is 596 times greater than that at 1 kHz.

The ME laminate composite can be used as a DC magnetic field sensor if the section in which *α*_ME_ changes sensitively and linearly according to the change in *H*_DC_ (e.g., the regions indicated in red in Figure 3a) is well defined. The length-to-width aspect ratio (*l*/*w*) of the FM layers can be controlled to adjust the *H*_DC_ of the maximum *α*_ME_ [22], i.e., to adjust the slope of the linear section and the magnetic field range to be detected. Furthermore, the ME laminate composite generates a voltage (*V*_AC_) under the *H*_AC_ (Equation (1)), and the *V*_AC_ is proportional to the *H*_AC_ (i.e., linearly varies with the proportional constant *t*_p_*α*_ME_). Therefore, the ME laminate composite can be used as an AC magnetic field sensor as well. The *H*_AC_ frequency of the sensor at which the enhanced *α*_ME_ is observed can be controlled by simply adjusting the length *l* of the sensor (i.e., adjusting the *f*_a_ of the sensor), and thereby, a highly sensitive AC field sensor for the desired frequencies can be implemented.

**Figure 4:**
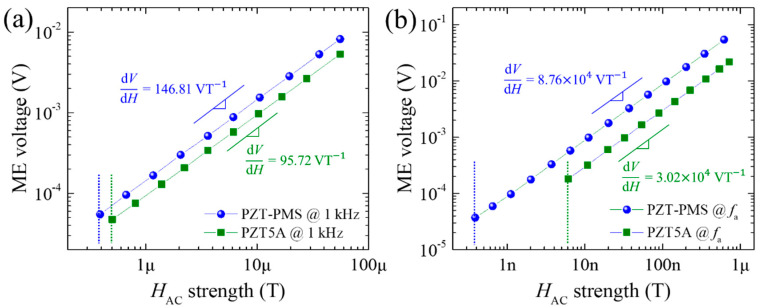
Magnetoelectric voltage output as a function of *H_AC_* of PZT-PMS and PZT5A sensors (FE dimensions: 13.5 mm (*l*) × 3.0 mm (*w*) × 1.0 mm (*t*)): (**a**) measured at 1 kHz and (**b**) measured at an anti-resonance frequency of the sensors. Slope values and dashed lines indicate magnetic field sensitivities and observed sensing limits, respectively.

Figure 4 presents the *V*_AC_ versus *H*_AC_ curves of the PZT-PMS and PZT5A sensors (FE dimensions: 13.5 mm (*l*) × 3.0 mm (*w*) × 1.0 mm (*t*)) obtained at 1 kHz and *f*_a_ of the sensors for evaluating the performance of the AC magnetic field sensors in the off-resonance and resonance modes, respectively. At 1 kHz, the PZT-PMS sensor displayed a linear relationship between the *V*_AC_ and *H*_AC_ at a *H*_AC_ greater than 390 nT, as shown in Figure 4a. At a *H*_AC_ less than 390 nT, the *V*_AC_ fluctuated so severely that it could not be measured as a constant value, indicating that the sensing limit of the PZT-PMS sensor was approximately 390 nT. The sensing limit of the PZT5A sensor was slightly larger than that of the PZT-PMS sensor. The magnetic field sensitivity (d*V*/d*H*, the slope of the linear section) of the PZT-PMS sensor (146.81 VT^−1^) was 1.5 times greater than that of the PZT5A sensor mainly owing to the greater *g*_31_ of the PZT-PMS. Meanwhile, in the resonance mode in Figure 4b, the sensing limit of the sensors was greatly improved: approximately 0.387 and 6.11 nT for the PZT-PMS and PZT5A sensors, respectively. Furthermore, the PZT-PMS sensor exhibited a significantly large sensitivity of 8.76 × 10^4^ VT^−1^, which is 2.9 times greater than that of the PZT5A sensor. This large difference in sensitivity values emphasizes the importance of the *Q*_m_ of the FE material when the sensor is operated in the resonance mode.

**Figure 5:**
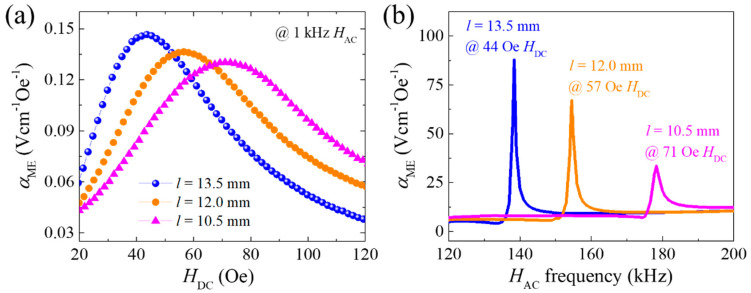
(**a**) Magnetoelectric voltage coefficient of PZT-PMS sensors with different lengths measured at 1 kHz as a function of *H*_DC_. (**b**) Magnetoelectric voltage coefficient spectra of the PZT-PMS sensors as a function of *H*_AC_ frequency. (FE cross-sectional dimensions: 3.0 mm (*w*) × 1.0 mm (*t*)).

As mentioned earlier, the length-to-width aspect ratio (*l*/*w*) of the FM layers can be controlled to adjust the *H*_DC_ of the maximum *α*_ME_. Figure 5a shows *α*_ME_ versus *H*_DC_ curves for the PZT-PMS sensors with various lengths and fixed width and thickness ((i.e., different *l*/*w*) measured at 1 kHz *H*_AC_. The *H*_DC_ of the maximum *α*_ME_ gradually increased with decreasing sensor length, resulting in a decrease in the slope of the linear section. The maximum *α*_ME_ also decreased slightly with decreasing sensor length, indicating that magnetostriction in the longitudinal direction of the FM layers became difficult due to the reduced *l*/*w*. In the resonance mode, the *H*_AC_ frequency of the maximum *α*_ME_ increased with decreasing sensor length, accompanied by a large reduction of the maximum *α*_ME_, as shown in Figure 5b. From these large reductions of the maximum *α*_ME_ in the resonance mode, it can be deduced that a small *l*/*w* causes a large mechanical loss in the FE layer.

**Figure 6:**
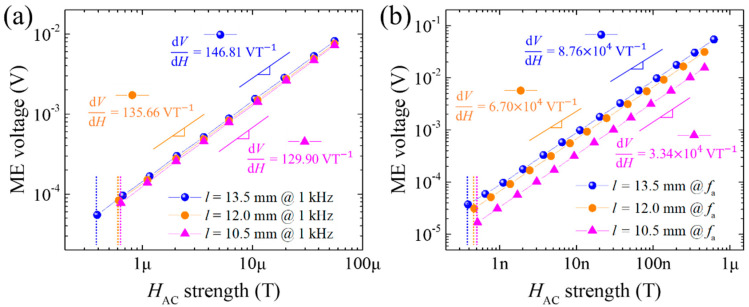
Magnetoelectric voltage output as a function of *H_AC_* of PZT-PMS sensors with different lengths (FE cross-sectional dimensions: 3.0 mm (*w*) × 1.0 mm (*t*)): (**a**) measured at 1 kHz and (**b**) measured at an anti-resonance frequency of the sensors. Slope values and dashed lines indicate magnetic field sensitivities and observed sensing limits, respectively.

The magnetic field sensitivity and sensing limit of the PZT-PMS sensors with different sensor lengths are displayed in Figure 6. Both in the off-resonance (1 kHz) and resonance (*f*_a_) modes, magnetic field sensitivity exhibited the same trend as the maximum *α*_ME_ observed in Figure 5, and the sensing limit was slightly increased with decreasing sensor length. Therefore, it is clear that a larger *l*/*w* is advantageous to obtain higher sensitivity and lower sensing limit under the fixed condition of the sensor thickness.

**Figure 7:**
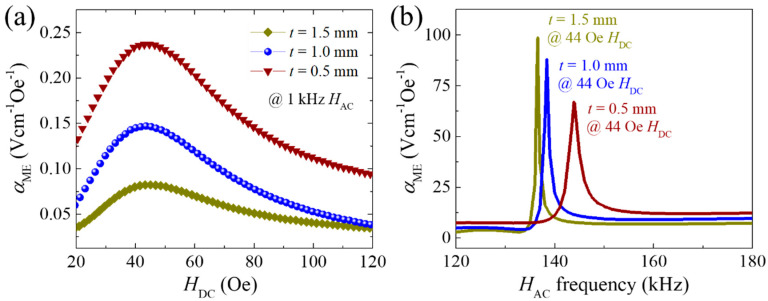
(**a**) Magnetoelectric voltage coefficient of PZT-PMS sensors with different FE layer thicknesses measured at 1 kHz as a function of *H*_DC_. (**b**) Magnetoelectric voltage coefficient spectra of the PZT-PMS sensors as a function of *H*_AC_ frequency. (FE areal dimensions: 13.5 mm (*l*) × 3.0 mm (*w*)).

Next, we investigated the effect of FE layer thickness (*t*) on the ME characteristics and magnetic field sensing performance. Figure 7a shows *α*_ME_ versus *H*_DC_ curves of the PZT-PMS sensors with various FE layer thicknesses, under fixed *l* and *w* conditions, measured at 1 kHz *H*_AC_. The Metglas FM layers of identical dimensions were applied to the PZT-PMS sensors; therefore, the *α*_ME_ values of the sensors were maximized at similar *H*_DC_ values. The maximum *α*_ME_ was greatly enhanced with decreasing *t*, exhibiting 0.236 Vcm^−1^Oe^−1^ when *t* = 0.5 mm, while it decreased to 0.084 Vcm^−1^Oe^−1^ as *t* increased to 1.5 mm. However, the maximum *α*_ME_ in the resonance mode exhibited a tendency opposite to that in the off-resonant mode, as shown in Figure 7b. The largest *α*_ME_ of 99.2 Vcm^−1^Oe^−1^ was obtained when *t* = 1.5 mm, and the sensor with *t* = 0.5 mm showed a reduced *α*_ME_ of 65.5 Vcm^−1^Oe^−1^.

The results in Figure 7 signify that the high thickness fraction of the FM layers are advantageous in the off-resonance mode, but is disadvantageous in the resonance mode for achieving high *α*_ME_. When *t* is small, magnetostriction of the FM layers can be readily generated, and the strain is effectively transferred to the FE layer. However, the electromechanical resonance of the FE layer is affected by structural damping, i.e., the resonance is attenuated if the motion of the FE layer is disturbed by the FM layers. Thus, a small *t* (or high thickness fraction of the FM layers) could lead to a large mechanical loss of the FE layer in the resonance mode.

**Figure 8:**
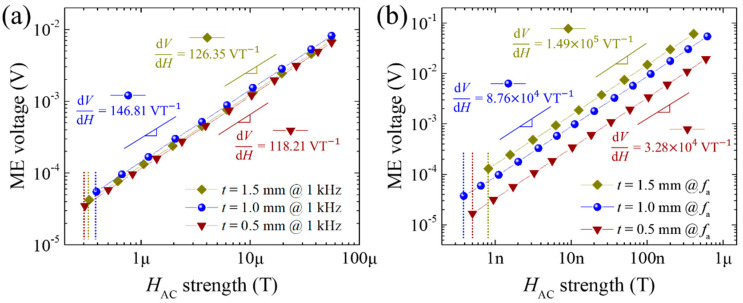
Magnetoelectric voltage output as a function of *H_AC_* of PZT-PMS sensors with different FE layer thicknesses (FE areal dimensions: 13.5 mm (*l*) × 3.0 mm (*w*)): (**a**) measured at 1 kHz and (**b**) measured at an anti-resonance frequency of the sensors. Slope values and dashed lines indicate magnetic field sensitivities and observed sensing limits, respectively.

Although the maximum *α*_ME_ values differed greatly as the *t* of the PZT-PMS sensors was changed (Figure 7a), sensors exhibited no significant difference in magnetic field sensitivity and sensing limit in the off-resonance mode, as shown in Figure 8a. When voltage monitoring is the basic sensing process of the magnetic field sensor system, the change in output voltage, not the *α*_ME_ value, should be high to increase the sensitivity. If magnetostriction strain of the same magnitude is transferred to the FE layer, the FE layer with larger *t* should generate a higher output voltage. However, the sensors with different *t* values exhibited similar sensitivities (or similar output voltages), implying that the magnetostriction of the FM layers decreased as *t* increased.

Unlike the off-resonance case, sensitivity showed a remarkable difference depending on the *t* of the sensor in the resonance mode, as shown in Figure 8b. Despite the smallest magnetostriction of the FM layers, the sensor with *t* = 1.5 mm exhibited the highest sensitivity of 1.49 × 10^5^ VT^−1^, whereas the sensitivity of the sensor with *t* = 0.5 mm, which is believed to generate the highest magnetostriction, was 4.5 times lower than *t* = 1.5 mm case. This result confirms that the *Q*_m_ at *f*_a_ (*Q*_m,fa_) of the sensor system predominates the magnetic field sensing performance in the resonance mode. The *Q*_m,fa_ values of the PZT-PMS sensors with *t* = 0.5, 1.0, and 1.5 mm were measured to be 71.4, 190.5, and 259.6, respectively. Even the *Q*_m,fa_ of the sensor with *t* = 1.5 mm was much smaller than that of the PZT-PMS material itself (*Q*_m,fa_ = 1783), indicating a large mechanical loss in the FE layer caused by the FM and bonding layers. This inevitable mechanical loss can be more serious when the sensor size is reduced, thus, the *Q*_m_ of the FE material should be as high as possible to achieve high sensitivity in the resonance mode. The PZT-PMS sensors with different *t* values exhibited very low sensing limits ranging from 0.387 to 0.816 nT at their *f*_a_.

The *α*_ME_, magnetic field sensitivity and sensing limit values of the ME composite sensors are summarized in Table 2. The data in Table 2 were obtained in the air at room temperature without using special shielding instruments. Nevertheless, the simple laminate composite with polycrystalline PZT-PMS ceramics exhibited a high sensitivity of 10^5^ VT^−1^ with a sub-nT sensing limit, thus demonstrating its feasibility as a small and cost-effective magnetic field sensor device.

## 4. Conclusions

In this study, the magnetic field sensing performances of FM/FE/FM laminate composites fabricated using PZT5A and PZT-PMS polycrystalline FE ceramics were evaluated and compared. The *g*_31_ of the FE layer affected the ME and magnetic field sensing performances of the laminate composites in the off-resonance mode. More importantly, the laminate composite sensor comprising the FE material with a high *Q*_m_ exhibited a large *α*_ME_ in the resonance regime, which resulted in a large magnetic field sensitivity in the sub-nT-level field range. The large length-to-width aspect ratio of the composite sensor was found to be desirable to reduce the mechanical loss in the FE layer. Furthermore, it was demonstrated that the higher the thickness fraction of the FE layer, the less mechanical loss of the sensor. The results of this study provide design guidelines for implementing high-sensitivity ME sensor devices from a material and device structure perspective.

## Figures and Tables

**Table 1:** Piezoelectric charge coefficient (*d*_31_), piezoelectric voltage coefficient (*g*_31_), electromechanical coupling coefficient (*k*_31_), mechanical quality factors at the resonance frequency (*Q*_m_ at *f*_r_) and anti-resonance frequency (*Q*_m_ at *f*_a_), dielectric constant (*ε*_33_^T^/*ε*_0_), and dissipation factor (tan *δ*) of 31-mode PZT-PMS and PZT5A ceramics.

	*d*_31_ (pCN^−1^)	*g*_31_ (×10^−3^ VmN^−1^)	*k*_31_	*Q*_m_ (at *f*_r_)	*Q*_m_ (at *f*_a_)	*ε*_33_^T^/*ε*_0_ (at 1 kHz)	tan *δ* (at 1 kHz)
PZT-PMS	−65.7	−16.8	0.33	1668	1783	442	0.002
PZT5A	−185.6	−11.0	0.37	71	101	1905	0.015

**Table 2:** Magnetoelectric voltage coefficient (*α*_ME_), magnetic field sensitivity, and sensing limit of PZT5A and PZT-PMS sensors.

FE Material	FE Material Dimensions (*l* × *w* × *t*, mm)	*α*_ME_ (Vcm^−1^Oe^−1^)		Sensitivity (VT^−1^)		Sensing Limit (nT)	
		@ 1 kHz	@ *f*_a_	@ 1 kHz	@ *f*_a_	@ 1 kHz	@ *f*_a_
PZT5A	13.5 × 3 × 1	0.096	30.1	95.72	3.02 × 10^4^	502	6.11
PZT-PMS	13.5 × 3 × 1	0.147	87.6	146.81	8.76 × 10^4^	390	0.387
	12.0 × 3 × 1	0.136	67.0	135.66	6.70 × 10^4^	613	0.466
	10.5 × 3 × 1	0.131	33.4	129.90	3.34 × 10^4^	641	0.514
	13.5 × 3 × 1.5	0.084	99.2	126.35	1.49 × 10^5^	334	0.816
	13.5 × 3 × 0.5	0.236	65.5	118.21	3.28 × 10^4^	307	0.504
